# Methodology for the development of a Canadian national EMS research agenda

**DOI:** 10.1186/1471-227X-11-15

**Published:** 2011-09-30

**Authors:** Jan L Jensen, Ian E Blanchard, Blair L Bigham, Katie N Dainty, Doug Socha, Alix Carter, Lawrence H Brown, Alan M Craig, Andrew H Travers, Ryan Brown, Ed Cain, Laurie J Morrison

**Affiliations:** 1 Emergency Health Services, 239 Brownlow Avenue, Suite 300, Dartmouth, NS, B3B2B2, Canada; 2 Dalhousie University Division of EMS, Halifax, NS, Canada; 3 Alberta Health Services, Emergency Medical Services, Calgary, AB, Canada; 4 Rescu, Li Ka Shing Knowledge Institute, St. Michael's Hospital, University of Toronto, Toronto, ON, Canada; 5 Hastings-Quinte EMS, Hastings County, ON, Canada; 6 James Cook University, Queenland, Canada; 7 Toronto Emergency Medical Services, Toronto, ON, Canada

## Abstract

**Background:**

Many health care disciplines use evidence-based decision making to improve patient care and system performance. While the amount and quality of emergency medical services (EMS) research in Canada has increased over the past two decades, there has not been a unified national plan to enable research, ensure efficient use of research resources, guide funding decisions and build capacity in EMS research. Other countries have used research agendas to identify barriers and opportunities in EMS research and define national research priorities. The objective of this project is to develop a national EMS research agenda for Canada that will: 1) explore what barriers to EMS research currently exist, 2) identify current strengths and opportunities that may be of benefit to advancing EMS research, 3) make recommendations to overcome barriers and capitalize on opportunities, and 4) identify national EMS research priorities.

**Methods/Design:**

Paramedics, educators, EMS managers, medical directors, researchers and other key stakeholders from across Canada will be purposefully recruited to participate in this mixed methods study, which consists of three phases: 1) qualitative interviews with a selection of the study participants, who will be asked about their experience and opinions about the four study objectives, 2) a facilitated roundtable discussion, in which all participants will explore and discuss the study objectives, and 3) an online Delphi consensus survey, in which all participants will be asked to score the importance of each topic discovered during the interviews and roundtable as they relate to the study objectives. Results will be analyzed to determine the level of consensus achieved for each topic.

**Discussion:**

A mixed methods approach will be used to address the four study objectives. We anticipate that the keys to success will be: 1) ensuring a representative sample of EMS stakeholders, 2) fostering an open and collaborative roundtable discussion, and 3) adhering to a predefined approach to measure consensus on each topic. Steps have been taken in the methodology to address each of these *a priori *concerns.

## Background

Evidence-based medicine is the "...conscientious, explicit, and judicious use of current best evidence in making decisions..."[1]. The practice of evidence-based medicine combines individual and organizational experience and expertise with the best available evidence to guide clinical care and system design. The challenge for many health disciplines, including emergency medical services (EMS), is the scarcity of research from which best evidence can be derived [2,3].

EMS has traditionally focused on emergency response to the sick and injured in the prehospital environment, and inter-facility transport. The last three decades have seen considerable expansion of the scope of practice of EMS personnel and the sophistication of EMS operations. EMS systems and paramedics are now seen as integral parts of the health care system, with their roles expanding to include not only emergency response and transport, but injury prevention and control, community health, public education, and emergency preparedness [3]. This expansion has occurred, for the most part, independent of any formal measurement and evaluation of outcome.

A foundation of research is required to support an evidence-based approach to prehospital care. While progress has been made in developing this foundation, EMS research is still in its infancy. The further development of Canadian EMS research has been identified by key stakeholders as a priority [3].

The research enterprise in EMS, like other health disciplines, is fraught with barriers and opportunities [4,5]. Other countries have recognized the value in systematically identifying barriers to, opportunities in, and priorities for EMS research. Both Australia and the United States have created research agendas in an effort to coordinate and focus resources to improve the EMS research enterprise. In 2002, a national two-day convention was held in Australia which included stakeholders such as ambulance authorities, universities, the professional college and others. They worked in large and small group sessions to identify research priorities, ways to encourage research, and the roles different organizations have in research projects [6]. In the same year, the United States *National EMS Research Agenda*, developed through multidisciplinary discussions and iterative expert writing and reviews, was published [4]. This seminal document identified five key barriers to the progress of research, and made recommendations to overcome each. The US agenda lead to the creation of a *National EMS Research Strategic Plan*, which identified priority areas for EMS research [7].

While the EMS systems of Australia and the United States share some commonalities with Canada, such as paramedic-based EMS systems, there are also many significant differences, such as how practitioners are trained, funding mechanisms both for practice and for research, and infrastructure. Research barriers, opportunities, and priorities may not be generalizable from these countries to Canada due to the unique constellation of factors that have a profound impact on the provision of EMS services and the research enterprise, and the time that has lapsed since the Australian and US agendas were published. To that end, we have undertaken the development of an EMS research agenda for Canada. Here we report our methodology with the intent that it may serve as an important starting point for other countries that are attempting to define their research agenda and improve the effectiveness and efficiency of their research enterprise.

## Methods/Design

To ensure that the Canadian National EMS Research Agenda is produced using an evidence- based approach that reflects the diversity of EMS systems in Canada, a three-phase mixed methods study has been designed. The project will consist of: 1) qualitative baseline interviews, 2) a facilitated roundtable discussion among key informants, and 3) a Delphi consensus survey. The results of each phase will inform subsequent phases.

### Stakeholders

The study team will develop a list of stakeholder categories of potential participants who may be able to provide important input to the study (Table 1). Purposeful sampling will be used to populate each category with approximately five potential participants. Additionally, one representative of each of the following national organizations will be invited: the EMS Chiefs of Canada, the Paramedic Association of Canada, the Canadian Association of Emergency Physicians (EMS Committee), the Society for Prehospital Educators of Canada, the Canadian Organization of Paramedic Regulators, and the International Association of Emergency Managers - Canada. Representation will be sought from all provinces, EMS system types (e.g., advanced and basic care, air and land ambulance) and professional types (e.g., paramedics and physicians). The total sample will include approximately 45 participants.

**Table 1:** Participant categories and definitions

Participant Category	Definition
Paramedic researcher	Paramedics who have dedicated time for research, or are regularly involved in conducting research studies

EMS physician researchers	Physicians who have dedicated time for research, and their research focus is EMS research

Researchers who use EMS data	Researchers who don't specialize in EMS, but use EMS data for their research

Paramedic educator	Paramedics who are employed by colleges, universities or EMS systems as educators of paramedic students or practicing paramedics

Front-line EMS providers	Field paramedics/communication officers/flight staff or others who primarily work in the ambulance, air medical transport or other out of hospital clinical settings.

EMS operations/management	Those who supervise or manage the operations of an EMS system

EMS regulators/colleges	Those who primarily work within a government organization that regulates an EMS system

EMS medical directors	Physicians who work in the role of medical director, overseeing clinical care in an EMS system

Potential participants will be emailed an invitation letter and participant response form. Of those participants who return the participant response form, a sub-sample will be contacted by email, specifically recruiting them to participate in the qualitative baseline interviews. All participants will be invited to participate in the roundtable discussion in St. John's, Newfoundland, Canada on June 8^th^, 2011 and in the Delphi consensus survey, which will be conducted after the results of the roundtable discussion are available.

### Ethics

Research ethics board approval was received for the qualitative interviews from St. Michael's Hospital, Toronto ON Canada (11-011c), and for the roundtable session and consensus survey from the Capital District Health Authority, Halifax NS Canada (CDHA-RS/2011-248).

For the telephone qualitative baseline interviews, informed consent will be verbally obtained by the interviewer (KD). For the roundtable discussion and consensus survey, participants will complete a written informed consent procedure prior to the roundtable discussion.

### Phase 1: QUALITATIVE BASELINE STUDY

#### Objective

The purpose of this phase is to gain a baseline understanding of the perspective of key stakeholders with regard to the landscape of EMS research in Canada in order to provide a framework for the roundtable discussion. Areas of interest include: the barriers and opportunities in EMS research; recommendations for enhancing the research enterprise; and topic areas within EMS research believed to be a high priority.

#### Design

A qualitative key informant interview study will be conducted, using one-on-one semi-structured telephone interviews with a sub-sample of the invited participants.

Ideally, the total sample will consist of 3-4 members of each key group, and invited organizations, for a total of ~ 20 participants. There will also be an attempt to have as diverse a representation from all provinces, EMS system types and professional types (e.g., paramedics, physicians, managers, researchers, etc.) as possible.

The sub-sample of invited participants will be sent an email which explains the qualitative study and includes a letter of information and invitation to participate. The lead investigator of the qualitative study (KD) will obtain verbal consent and conduct the interviews with those who volunteer. The purposeful sampling technique will be complemented with snowball sampling by asking interviewees to identify individuals who they feel should be added to the sample.

An interview guide will be developed, based on the information from the literature and the areas of interest for the study. All interviews will be conducted by telephone for consistency, and the interviewer will use the study guide along with additional probing questions to facilitate the interviews. Interviews will be audio recorded for verbatim transcription and analysis and the interviewer will take supplemental field notes during the conversation. Data collection will be considered complete once saturation is reached; that is, when little new information is expected to be learned from further interviews [8].

#### Data Analysis

Two investigators (KD and BB) will conduct the qualitative data analysis using a constant comparative method [9]. Analysis will begin with both investigators reading through transcripts as they are completed, in order to gain an understanding of the issues discussed and to develop a preliminary categorization scheme. Categories will be added to the scheme as new transcripts are reviewed. Each transcript will then be read a second time, and participant statements will be coded according to the categories using NVivo qualitative analysis software (QSR, Doncaster Victoria Australia). The two investigators will compare their independent analyses for the first four transcripts, and will discuss differences in coding and if new categories should be added. After all transcripts are coded, the two investigators will review the coding scheme to identify key emergent themes and begin to interpret how the data relates to these key issues and the Research Agenda objectives. The investigators will also note if any relationships exist between participant location, position or involvement in research and the key themes identified.

A summary of the results of the analysis will be shared with all interviewees to engage them in any clarifications required to ensure the summary document accurately includes their input to the study [10]. The final summary document will then be presented to the study team as a guide for the organization of the roundtable discussion.

### Phase 2: ROUNDTABLE SESSION

#### Objectives

The purpose of the roundtable key informant discussion is to gather a purposeful sample of key stakeholders (the participants) from across Canada to discuss the four study objectives: to further identify and discuss barriers and opportunities for Canadian EMS research, make recommendations for the future, and identify research priorities.

#### Design

Participants will be provided with preliminary results from the baseline qualitative study prior to the roundtable session. An in depth review of the findings will also be presented at the session. In addition, a US EMS researcher will present information on his experiences as a member of the team that set the US EMS Research Agenda (LB) [4].

The roundtable session will be based on the methodology of a successful meeting that set a Canadian EMS agenda for Patient Safety in 2010 [11]. The roundtable will consist of facilitated small and large group sessions, moderated by a professional facilitator. Each session will focus on one of the study objectives (Table 2).

**Table 2:** Agenda for roundtable discussion

Welcome, Introductions, Setting the Context

Experiences from the US EMS Research Agenda

Results of the Baseline Qualitative Interviews

Objective 1. Barriers to EMS Research (Small Group Session and Large Group Reporting)

Objective 2. Current System Strengths & Existing Opportunities for EMS Research (Small Group Session and Large Group Reporting)

Objective 3. Recommendations for the Future of Canadian EMS Research (Small Group Session and Large Group Reporting)

Objective 4. Priorities in Canadian EMS Research (Small Group Session and Large Group Reporting)

Large Group Debrief and Discussion, Roundtable Wrap-Up

Participants will be purposefully placed into small groups. Each group will contain a mix of participants from different participant categories, with careful attention paid to creating groups that are geographically diverse. A facilitator will be assigned to each table, and will lead the small groups by encouraging participants to openly discuss their thoughts on each study objective. Small group facilitators will move discussions forward by using probing questions to explore topics identified by the participants. Small group facilitators will meet with the professional facilitator prior to the roundtable session to ensure that an appropriate and consistent approach is taken to the small group facilitation. The professional facilitator and two group facilitators will also circulate amongst the small groups during the session to listen to the conversations and ensure a uniform approach is being taken by all small group facilitators.

#### Data Collection

Each participant will complete the written informed consent procedure, a disclosure of conflict of interest form and a short demographic questionnaire, for the purpose of accurately reporting sample characteristics.

For each of the four objectives, participants will break into their assigned small groups. All small groups will be provided with flipcharts, where they will record topics discussed related to each objective. In addition, every participant will be provided worksheets for each study objective, in which they can list all topics they feel are important for each objective. These worksheets will be collected. After the completion of each small group session, the large group will re-convene and share topics and items that emerged during small group discussion. An investigator will record all topics reported verbally.

#### Data Compilation

After the completion of the roundtable, two investigators (JJ & RB) will review all the recorded information from the day, and will organize the topics into the four project objectives. Duplicate items in a category will be removed. Original participant wording will be maintained as much as possible, for use in the subsequent Delphi consensus survey [12].

### Phase 3: DELPHI CONSENSUS SURVEY

#### Objective

The purpose of the Delphi consensus survey is to identify, using participant consensus, the most important topics in each of the four study objectives.

#### Design

Delphi studies are frequently used in healthcare and other industries [13] to achieve consensus among a group of experts on a particular topic. This is accomplished through anonymous iterative surveys in which participants are asked to score items [12,14].

Within four weeks of the roundtable session, participants will be emailed a link to an online survey site. Each survey round will be open for five working days. In the first round, topics identified in the literature synthesis, qualitative interviews and roundtable session will be listed for each study objective (barriers, opportunities, recommendations and priorities). An additional text box will be provided for respondents to enter any further topics, thoughts or elaborations they have. Participants will score each topic on a Likert scale (1 = not important, 2 = not very important, 3 = possibly important, 4 = important, 5 = extremely important).

In the second and third rounds, the mean scores for each topic and the participant's own score will be available for review (i.e., each participant will see their own score for each topic and all participants will see the group mean scores for each topic). Participants will be able to re-score each topic, or keep the score they assigned in the previous round. As consensus is reached on the 'importance' [13] (or lack thereof) of individual topics, they will be removed from the Delphi survey. In the second and third rounds, participants may enter new topics into a free text box. The survey will be re-sent to a maximum of four rounds, to avoid sample fatigue.

Research Agenda participants will follow the Delphi technique to achieve consensus on the most important topics or items for each Research Agenda objective based on information gathered during the interviews and roundtable discussions.

#### Data Analysis

Data from each round of the Delphi survey will be downloaded from the Opinio survey tool (Objectplanet, Oslo, Norway) into a Microsoft Excel spreadsheet (Redwood, CA, USA), in which descriptive analysis of panel characteristics, categorization of free text, and analysis (mean scores and level of consensus) of each topic in each round will be conducted.

In Delphi surveys, it is essential to define consensus *a priori *[13]. For each topic (within each study objective) we will consider consensus to be achieved for the most important items if 80% of the participants scored the theme as 4 ('important') or 5 ('extremely important'). These topics will be removed from the list in subsequent rounds. In a similar fashion, consensus for the least important topics will be considered achieved if 80% or more of the participants score any topic a 1 ('not important') or a 2 ('not very important'). For each round, analysis for consensus will be conducted for the entire panel, and also for participant stratifications of the panel (e.g., paramedics, physicians, EMS managers, etc.).

Response rates for each round will be reported, as well as descriptive statistics of the participant demographics.

### Integration of Findings

To achieve the objectives of the Canadian National EMS Research Agenda a mixed methods approach will be used. This approach of collecting both qualitative and quantitative data to answer one research question is growing in popularity among researchers and funding agencies [15]. An essential component of mixed methods studies is effective integration of data; otherwise the project is essentially two independent studies of the same topic [16]. In this project, each phase of the study will inform the next stage, and the results will be integrated using triangulation, a process that contributes to the validity of the results [17]. During the design phase of this study, the study team established that the topic must be explored qualitatively, to learn more of the barriers and opportunities to Canadian EMS research - a previously unstudied topic. The qualitative data will be analyzed, and the results will then inform the roundtable discussion. The topics discussed during the roundtable will be entered into the quantitative Delphi consensus survey, which will then be analyzed. Data from all phases of the study will then be triangulated by two investigators (JJ and KD) [17]. The triangulation will consist of the following steps, performed independently by each researcher: sorting (reviewing the results and identifying prevalent themes in both the qualitative interviews and the consensus survey), convergence coding (was there full agreement, partial agreement, silence (i.e., one set of results addresses a theme, but it does not appear in the other set of results), or dissonance between each set of results), and comparison of triangulation findings between each researcher [17]. The final step of the triangulation protocol is providing feedback of the triangulation results to the study team. Through this process, convergent themes that appear to be important in both sets of results, silent themes and dissonant themes are identified. This information will allow the investigators to gain a greater understanding of the results and the research topic.

The final report will include the results of the qualitative findings from the baseline interviews, quantitative results from the roundtable and Delphi consensus survey, and the results of the triangulation exercise. The integration of these results will form the Canadian EMS Research Agenda.

### Knowledge Translation Plan

**Figure 1:**
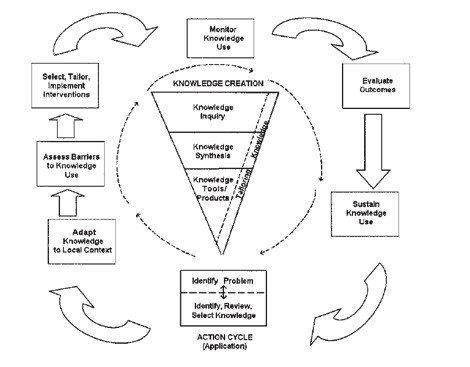
**Model of Knowledge to Action (18)**.

Graham et al (2006) coined the term 'knowledge to action' to describe the meaning and components of the knowledge translation process [18]. Knowledge translation is composed of two major processes: knowledge creation and the action cycle, in which the new or refined knowledge is implemented. The action cycle consists of the following phases: identifying a problem and the relevant knowledge; adapting the knowledge to the local context; assessing barriers (and enablers) to using the knowledge; implementing tailored interventions to promote use of the knowledge; monitoring knowledge use; evaluating the outcomes of using the knowledge and sustaining ongoing knowledge use (Figure 1).

#### Dissemination Strategies

A multi-faceted dissemination plan targeted at stakeholders will be executed at the completion of the Agenda project. The primary means of conveying the results of the study will be through publication of peer-reviewed manuscripts in journals that appeal to the target scientific and provider audience. Second, submissions will be made to prominent EMS trade journals targeting front-line paramedics, medical directors and EMS operators. Third, presentations at regional, provincial, and national EMS conferences will be used to communicate the results to attendees. Lastly, key organizations will be notified of the results and asked to convey them to their members through free electronic media and posting of the results on their free-access websites. As well the research agenda will be circulated to potential granting agencies, foundations, industrial and academic partners and follow up meetings with these constituents will enable full discussion and identify opportunities to lobby for change, influence priority and new funding initiatives.

Once the Agenda has been widely disseminated in Canada, it is expected it will serve as a guide for EMS stakeholders to implement a local and national strategy to address barriers to research and to maximize on existing and future opportunities. It may be used to inform budget planning and grant allocation and human resources for research, and to lobby for change in the way funding is directed in existing funding agencies. The implementation strategy is likely to include efforts to develop and protect researchers to conduct studies on questions found to be important, and will ignite collaboration across the country, which will allow for studies with sample sizes sufficient to answer vital questions.

#### Potential Challenges in Knowledge Translation

Once this study is complete, and results are disseminated through the mediums described above, we anticipate challenges in the following components of the knowledge translation action cycle: adaptation to the local context, monitoring knowledge use, and evaluating the outcomes of using the knowledge. EMS systems in Canada have had variable research experiences, ranging from no exposure to studies to being involved with the conduction of randomized controlled trials. Because of this variability, the implementation of the Canadian EMS Research Agenda will need to be adapted to individual services and settings. Local research stakeholders will need to review the Agenda and determine which barriers, opportunities, recommendations and research priorities make the most sense to adopt. The extent of implementation of the agenda will be determined through surveys and networking events, which will provide opportunities to capitalize on funding and partnership opportunities to conduct research. The ultimate goal of the Agenda is to improve patient care and EMS system delivery through the creation and use of meaningful and high quality EMS research. Possible benchmarks include grant funding acquired, publications, clinical outcomes (such as changes in survival), cost savings, and the number of EMS systems actively involved in research.

## Discussion

In this comprehensive mixed methods study, we anticipate the keys to success will be: 1) ensuring a representative sample of EMS stakeholders, 2) fostering an open and collaborative roundtable discussion, and 3) adhering to a predefined approach to measure consensus on each topic. Steps have been taken in the methodology to address each of these *a priori *concerns.

The findings of this study will provide leaders in EMS administration, regulation, medical direction, academia, clinical practice, government funding agencies, foundations and industrial partners with critical evidence to further strengthen Canadian EMS research.

## Abbreviations

EMS: emergency medical services

## Competing interests

JLJ and AC receive salary support from Emergency Health Services to conduct EMS research. IEB receives salary support from Alberta Health Services - EMS to conduct EMS research. EC receives salary support from Dalhousie University to direct and conduct EMS research. AMC receives salary support from the City of Toronto to conduct research and has received travel support from ZOLL Medical. KND receives salary support to conduct research from grant funding. LJM is an investigator with the Resuscitation Outcomes Consortium (ROC). ZOLL Medical Inc, Phillips and Medtronic have a partnership agreement with the NIH, which sponsors ROC. Their agreement pertains to the provision of equipment and software to the participating EMS and Fire Services. A portion of LJM's salary support is provided by the NIH Grant that supports the U of T ROC Regional Coordinating Centre. LJM holds grants from Canadian Institute of Health Research, Heart and Stroke Foundation Canada, American Heart Association, Laerdal Medical Foundation and the Ontario Ministry of Health. No other author has a financial or academic conflict of interest to disclose in regard to this study topic.

## Authors' contributions

JLJ, IEB, BLB and DS developed the research concept and plan. JLJ and KD obtained research ethics approval. JLJ, IEB, BLB, DS obtained funding. JLJ, IEB, BLB, DS, KD and RB recruited and enrolled participants. All authors contributed substantially to the design and methodology of this study and to the writing and critical editing of this manuscript, and intend to remain significantly involved in the study until completion. All authors have read and approved the final manuscript.

## Pre-publication history

The pre-publication history for this paper can be accessed here:

http://www.biomedcentral.com/1471-227X/11/15/prepub
